# Rerupture of nonparasitic liver cyst treated with cyst fenestration: a case report

**DOI:** 10.1186/s40792-015-0075-8

**Published:** 2015-09-02

**Authors:** Kentaro Inoue, Tomohiro Iguchi, Shuhei Ito, Takefumi Ohga, Tadahiro Nozoe, Ken Shirabe, Takahiro Ezaki, Yoshihiko Maehara

**Affiliations:** Department of Surgery and Science, Kyushu University, 3-1-1 Maidashi, Fukuoka, Japan; Department of Surgery, Fukuoka Higashi Medical Center, Koga, Japan

**Keywords:** Nonparasitic liver cyst rupture, Cyst fenestration, Acute abdomen

## Abstract

We herein describe a case involving spontaneous rerupture of a nonparasitic liver cyst successfully treated with cyst fenestration and an omental flap. A 59-year-old Japanese woman was transferred to our hospital for evaluation of acute abdominal pain. She had a history of conservative treatment with antibiotics for spontaneous rupture of a liver cyst 1 month previously. On arrival, she exhibited abdominal tenderness and muscular defense. Enhanced computed tomography showed ascites and a large ruptured hepatic cyst (diameter of 10 cm). We diagnosed rerupture of a liver cyst and performed laparotomy for cyst fenestration and intraperitoneal drainage. During the operation, we found the perforation site on the ventral side of the cyst and brown, muddled ascitic fluid. Cholangiography showed no bile leakage on the inner wall. Pathological investigation revealed no evidence of malignancy. The patient recovered without any adverse events and was discharged on postoperative day 8. No recurrences or complications occurred for 2 years.

## Background

A nonparasitic liver cyst (NLC) is a common benign liver disease. It is potentially asymptomatic and is often incidentally diagnosed with abdominal imaging such as ultrasonography or computed tomography (CT). With the advancements and spread of these abdominal imaging techniques, NLCs are becoming more frequently detected and have been found in approximately 5 % of the population [1]. In many cases, an NLC is asymptomatic and is conservatively followed up without treatment. However, NLCs are sometimes associated with various complications such as rupture, infection, hemorrhage, obstructive jaundice, portal hypertension, and pulmonary embolism. These complications occur in less than 5 % of all patients with NLC [2].

We herein describe a rare case of spontaneous rerupture of an NLC that had become exacerbated after conservative treatment and was successfully treated with surgical fenestration.

## Case presentation

**Fig. 1:**
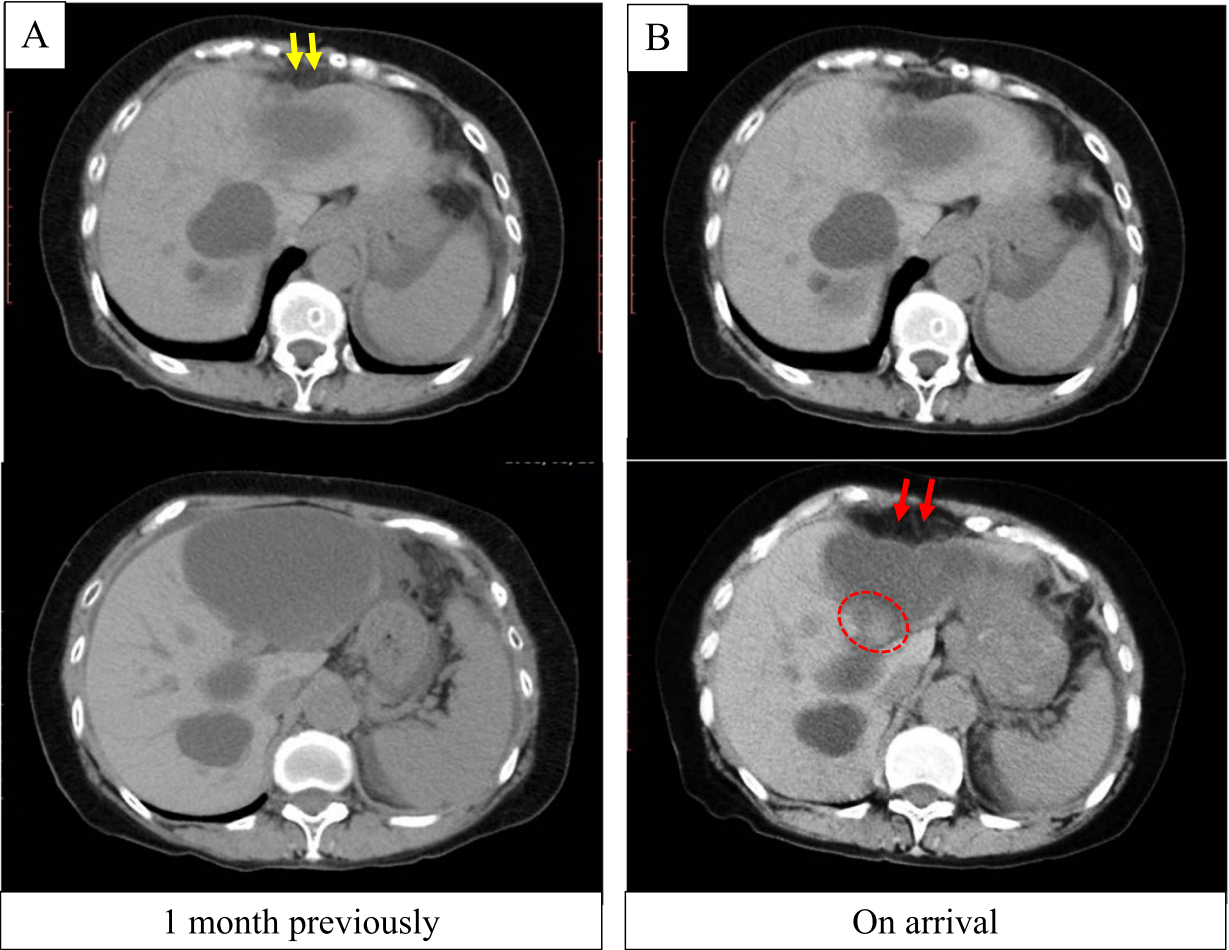
CT images of progression of hepatic cyst rupture. **a** CT image 1 month before presentation to our hospital. The largest cyst showed an irregularly shaped wall on the ventral side (*above*, *yellow arrows*). At that time, the caudal part of the cyst kept circular (*below*). Some ascitic fluid was found around the spleen. **b** CT image on arrival to our hospital. Volume of the irregularly shaped cyst had obviously decreased (*red arrows*) and was present within a relatively high dense lesion (*red circle*)

A 59-year-old Japanese woman was transferred to the emergency unit of our hospital for evaluation of acute abdominal pain. She had a history of conservative treatment for a spontaneous NLC rupture 1 month previously in another hospital (Fig. 1a).

On examination, she had a pulse rate of 115 beats/min, blood pressure of 112/68 mmHg, and no fever. Her abdomen was flat but hard and painful. She also exhibited obvious tenderness and muscular defense upon arrival. Blood tests revealed acute inflammation and anemia (Table 1). The levels of the tumor markers carcinoembryonic antigen and carbohydrate antigen 19-9 were within normal limits. Enhanced CT showed hepatic cysts and ascites. The largest cyst was found on the lateral segment; it exhibited an irregularly shaped surface and was present within a partially high dense lesion (Fig. 1b). The cyst volume had obviously decreased during the 1-month period before presentation to our hospital (Fig. 1, below). However, no neoplastic features such as thickened walls, papillary projections, or calcifications were found. The ascitic fluid collected by abdominal puncture was brown and muddled. The bilirubin level of the ascitic fluid was normal; however, the neutrophil and hemoglobin levels were high. Bacterial culture of ascetic fluid was negative (Table 2).

**Table 1:** Blood examination on arrival

White blood cells	17400	/μl
Neutrophil	89.8	%
Hemoglobin	10.7	g/dl
Platelets	247,000	/μl
Albumin	4.0	g/dl
Total bilirubin	0.53	mg/dl
Lactate dehydrogenase	255	IU/l
Aspartate aminotransferase	26	IU/l
Alanine transaminase	24	IU/l
Alkaline phosphatase	298	IU/l
Creatinine	0.5	mg/dl
C-reactive protein	0.26	mg/dl
Carcinoembryonic antigen	2.5	ng/ml
Carbohydrate antigen 19-9	<2.0	U/ml
α-fetoprotein	4.9	ng/ml

**Table 2:** Examination of ascitic fluid on arrival

Property	Brown and slightly muddled	
Cell counts	43980	/μl
Neutrophils	88	%
Total bilirubin	<0.01	mg/dl
Hemoglobin	1.0	g/dl
Bacterial culture	Negative	

Based on the patient’s clinical course and investigation findings, we diagnosed panperitonitis associated with rerupture of the liver cyst and accompanied by hemorrhage. Laparotomy was performed for cyst fenestration and intraperitoneal drainage.

**Fig. 2:**
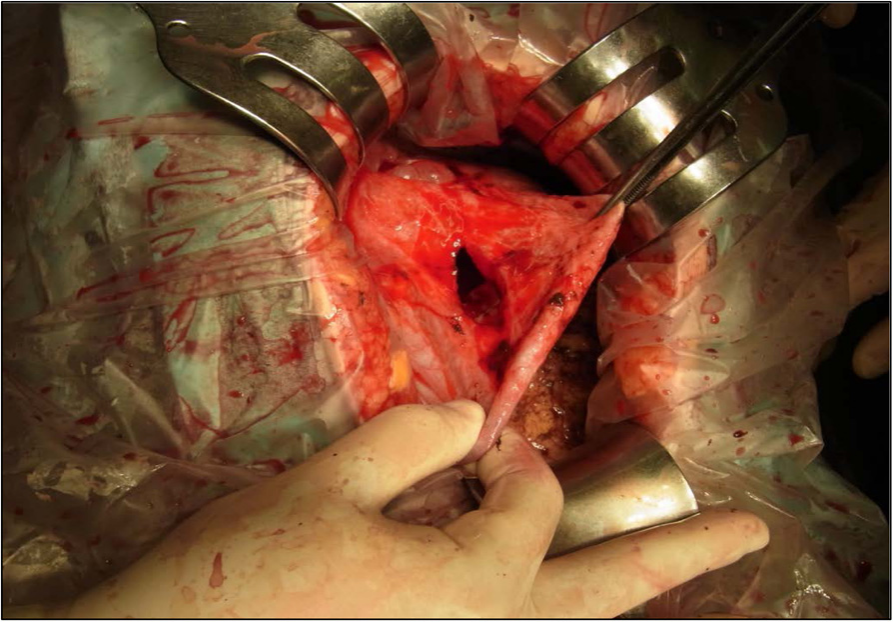
Perforation lesion of hepatic cyst. The perforation lesion was on the ventral side of the cyst. The lesion was approximately 3 cm, and the cyst wall was fibrous

**Fig. 3:**
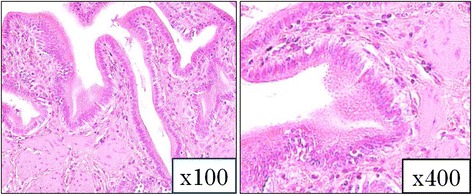
Pathological examination of cyst wall. Only fibrous connective tissue covered with simple cuboidal epithelium was observed; no evidence of malignancy was present

During the operation, we found the perforation site on the ventral side of the cyst (Fig. 2). The perforation was approximately 3 cm, and the cyst wall was fibrous. Although no obvious hematoma was detected, approximately 600 ml of ascitic fluid was found. The ascitic fluid was brown and slightly muddled. No nodules or other specific findings, indicating signs of malignancy, were found. We resected the ventral wall of the cyst followed by cholecystectomy and cholangiography. Cholangiography showed no bile leakage on the inner wall. We performed cyst argon beam coagulator ablation of the inner wall and covered the site with an omental transposition flap. The patient tolerated these procedures well and was transferred to the intensive care unit in a hemodynamically stable condition. Pathological examination showed only fibrous connective tissue covered with simple cuboidal epithelium; there was no evidence of malignancy (Fig. 3). The patient received antibiotics (PIPC/TAZ) until postoperative day 5. She recovered without any adverse events and was discharged on postoperative day 8. She was in good condition without recurrent symptoms 2 years postoperatively.

## Conclusions

Rupture of parasitic liver cysts, which are mainly caused by the *Echinococcus* species, is a well-known complication of such cysts and is often reported as hydatid cyst rupture [3, 4]. In contrast, rupture of NLCs is highly rare. The frequency is unknown, but Morgenstern [5] stated that only four cases of rupture are present among approximately 250 reports of solitary NLC published before 1958. In our computerized search of English-language reports of NLC rupture published from 1959 to 2013, we identified only 17 publications describing NLC rupture (Table 3) [3–19]. The causes of NLC rupture are variable and include infection, trauma, iatrogenic injury, and spontaneity [11, 16, 20]. In the current report, we presented a case of the second rupture without a specific cause such as infection or trauma after previous conservative treatment. The patient had acute abdomen and signs of preshock on arrival; clinical investigations showed mild anemia, acute systemic inflammation, and muddy ascitic fluid. The preoperative CT showed an irregularly shaped NLC with a high dense lesion. Therefore, we diagnosed the spontaneous rerupture of the NLC with hemorrhage and performed acute surgery. As intraoperative findings, no obvious hematoma was detected. However, comparing with the previous reports in Table 3, brown muddled ascites indicated the presence of hemorrhage. Therefore, in our case, the slight bleeding in the ruptured NLC could exist, and it might be the reason why the patient exhibited the acute abdomen.

**Table 3:** Review of nonparasitic liver cyst rupture

Year	Reference	Age	Sex	Symptoms	Peritoneal irritation	Cyst (cm)	Location (segments)	Ascites	property of ascites	Hemorrhage	Emergency procedures	Treatment	Outcome
2014	Our case	59	F	Acute abdominal pain	Yes	10	Left	Yes	Brown and slightly muddled	No active bleeding	Yes	Laparotomy and cyst fenestration	Uneventful
				Tenderness and muscular defense								Placing omentum over the ruptured cyst	
2013	Marion	37	F	Pain in the right hypochondrium	No	18	Right lobe S4	Yes	Hemoperitoneum blood clots	Yes	Yes	Cystectomy	Uneventful
				Tenderness in the right subcostal region									
				Pallor									
				Dyspnea									
2010	Ueda	64	F	Right upper quadrant pain	No	10	Right lobe	Yes	Serous brown	No	No	Percutaneous aspiration	Uneventful
												Injection of minocycline hydrochloride	
2010	Miliadis	70	M	Sudden diffuse abdominal pain	Yes	13	Right lobe	Yes	Opaque-yellowish peritoneal fluid	Unknown	Yes	Deroofing of the cyst	Uneventful
				Diffuse guarding									
												Omentoplasty	
				Rebound tenderness								Cholecystectomy	
2007	Salemis	50	M	Sudden severe abdominal pain	Yes	17	Left lobe	Yes	Unknown	Unknown	Yes	Wide excision of the cyst	Uneventful
				Nausea									
												Running locking suture along the edge of the resected cyst wall	
				Vomiting									
				Diffuse tenderness									
				Rebound tenderness									
2005	Cheung	73	F	Sever abdominal pain	Yes	17	Right lobe	Yes	Blood stained	Yes	Yes	Laparoscopic deroofing of ruptured cyst	Good condition
2003	Shutsha	67	F	Sudden sharp abdominal pain in the right upper abdomen after coughing fit	No	Unknown	Multiple	Yes	Unknown	No	-	None because abdominal pain spontaneously disappeared within 2 days	Good condition
2003	Kanazawa	78	M	Sudden onset of sever right hypochondralgia	No	Unknown	Right lobe	Yes	Dark, bloody-colored pus	Yes intracystic	No	Antibiotics	Good condition
												Drainage and alcohol injection	
				Tenderness in the right hypochondral region without muscle defense									
2002	Ishikawa	42	F	Discomfort in upper abdomen	No	10	S4 and S5	Yes	Muddy, dark brown	Yes	No	Transcatheter arterial embolization (TAE)	Uneventful
						13 after TAE						Drainage	
												Cystectomy	
2002	Carel	76	M	Progressive abdominal pain	Yes	9	Right lobe	Yes	hemoperitoneum	Yes	Yes	Laparotomy	Death 4 weeks after admission due to complications (hemodynamic instability, arrhythmias, bacterial pneumonia)
				Severe tenderness								Placing omentum over the ruptured cyst	
				Diffuse rebound pain									
1999	Yamaguchi	61	M	Spontaneous pain in the right upper quadrant of the abdomen	Yes	13	Left and S5	Yes	With blood clot	Yes	no	Hepatectomy due to involving anterior branch of right portal vein	Uneventful
									No preoperative investigation				
				Tenderness									
				Muscular defense									
1999	Payatakes	62	unknown	Acute right upper quadrant abdominal pain	-	9.5	Right	-	-	-	-	Partial excision	Symptom free
												External drainage	
1989	Akriviadis	48	F	Sever epigastric pain	-	Unknown	Left	-	-	-	No	Conservatively	Uneventful
1988	Ayyash	36	M	Sudden epigastric pain	-	4	Right	-	-	-	No	Conservatively	Uneventful
				Vomiting									
1974	Brunes	54	F	Diffuse abdominal pain	-	25	Left	-	-	-	-	Partial removal of the ruptured cyst	Symptom free
1972	Russell	68	M	Sudden severe abdominal pain	-	12	Left	-	-	-	-	Left lobectomy	Uneventful
1960	Johnston	82	F	Right-sided abdominal pain	-	15	Right	-	-	-	-	Catheter drainage	Died on third postoperative day
				Vomiting									
1959	Morgenstern	56	F	Sudden severe abdominal pain	Yes	35	Left	Yes	Dark greenish brown	Unknown	Yes	Lobectomy	Uneventful
				No vomiting								Decompressing cholecystostomy	

In general, treatment options for symptomatic NLCs include surgical procedures and conservative management such as percutaneous needle aspiration and drainage [21]. Percutaneous needle aspiration is a less invasive intervention than a surgical operation and can also be used to examine the properties of the cyst contents. However, it is associated with high relapse rates of >80 %. This high recurrence rate can be decreased by about 20 % when percutaneous needle aspiration is combined with alcohol minocycline chloride or tetracycline chloride injection [22, 23]. In our case, the patient underwent the only conservative management after the initial rupture of NLC without any adjunctive procedures. This could be one reason why the rerupture occurred. With respect to surgical management, open or laparoscopic cyst fenestration, also termed deroofing, is a definitive and widespread treatment [24]. Argon beam coagulation and electrocoagulation to destroy the remaining epithelium and placement of an omental transposition flap after fenestration can also contribute to reduced relapse rates [25]. Complete cyst excision and partial hepatectomy have been performed in some cases because of concern regarding malignancy. However, these operations are highly invasive and almost unacceptable for benign diseases despite the fact that the reported recurrence rate is 0 % [11, 25]. Therefore, in the present case, we performed emergent laparotomy, cyst fenestration, argon beam coagulation of the remaining cyst wall, and placement of an omental transposition flap.

The optimal treatment strategy and surgical indications for NLC rupture are not clearly defined. Conservative management including percutaneous drainage might be useful for cases without critical features such as signs of peritoneal irritation and shock [7]. However, as shown in the current case, rerupture of an NLC after conservative treatment should be considered. In terms of curability, the risk of relapse, and the possibility of other complications such as hemorrhage, cyst fenestration might be more favorable in most cases.

In conclusion, rupture of an NLC is a highly rare complication but can be a cause of the acute abdomen. Clinical observation and conservative treatment including percutaneous needle aspiration and drainage might be beneficial; however, careful consideration of the optimal therapy and performance of close follow-up are necessary owing to the possibility of relapse.

## Consent

Written informed consent was obtained from the patient for publication of this case report and any accompanying images. A copy of the written consent is available for review by the Editor-in-Chief of this journal.

## Abbreviations

CT: computed tomography
NLC: nonparasitic liver cyst
